# Providing easier access to community‐based healthcare for people with joint pain: Experiences of delivering *ESCAPE‐pain* in community venues by exercise professionals

**DOI:** 10.1002/msc.1584

**Published:** 2021-08-10

**Authors:** Michael Hurley, Helen Sheldon, Margaret Connolly, Andrea Carter, Rachel Hallett

**Affiliations:** ^1^ Health, Social Care and Education St George's University of London and Kingston University London UK; ^2^ Musculoskeletal Programme Health Innovation Network London UK; ^3^ Department of Psychology Open University Milton Keynes UK

**Keywords:** community‐based, *ESCAPE‐pain*, joint pain

## Abstract

**Background:**

Joint pain adversely impacts the physical, mental, socioeconomic and emotional wellbeing of many millions of people*. Enabling Self‐management and Coping with Arthritic Pain using Exercise, ESCAPE‐pain*, is a rehabilitation programme that reduces joint pain and its impact. The programme is usually delivered in clinical settings by physiotherapists but delivering it in community venues would improve access greatly.

**Aim:**

To explore the feasibility of delivering *ESCAPE‐pain* in community venues, and the experiences of organisations and facilitators delivering it.

**Methods:**

Semi‐structured interviews were conducted with managers of 17 community organisations and 10 facilitators.

**Results:**

People were happy to attend *ESCAPE‐pain* delivered by exercise professionals at community venues, which they found convenient and valuable. It expanded community organisation's offer to older people, utilised their facilities off‐peak and advanced facilitator's personal and professional development. Recruitment onto the programme was easiest where there were good links with local clinical providers. Although collecting outcome data was burdensome it demonstrated the programme's effectiveness to commissioners. Some clinical commissioners contracted community organisations to deliver *ESCAPE‐pain* reducing their costs and freeing up clinical facilities. Organisations also financed *ESCAPE‐pain* by charging participants a nominal fee for the programme, post‐programme classes to support participants remain active and/or a membership fee.

**Conclusions:**

*ESCAPE‐pain* delivered in community venues facilitated access to better care and on‐going support. Partnerships between healthcare commissioners and community providers maximised efficient use of their facilities and resources and fulfilled national policy of encouraging self‐management of long‐term conditions in the community.

## 1 INTRODUCTION

Worldwide osteoarthritis (OA) is a leading cause of joint pain and disability, impairing mobility, physical and psychosocial health and wellbeing and quality of life (Hunter et al., 2014; Vos et al., 2020). In the UK OA affects nearly 9 million people, whose annual health and social care costs are almost £5 billion–the fourth largest source of expenditure by the NHS (NHS England, 2015; Versus Arthritis, 2019). These personal and societal costs are increasing rapidly as more people live longer, but are less active and obesity increases, as inactivity and obesity are important risk factors for developing OA (Hunter & Bierma‐Zeinstra, 2019; Public Health England, 2018; Versus Arthritis, 2019). Consequently, joint pain due to OA a major and rapidly growing public health problem (Cross et al., 2014; Public Health England, 2018; Versus Arthritis, 2014; Vos et al., 2020), that will have been exacerbated by the COVID19 pandemic.

All international management guidelines (National Institute for Health & Clinical Excellence, 2014; Rausch Osthoff et al., 2018) recommend physical activity to reduce joint pain and mitigate it's physical and psychosocial impact (Hurley et al., 2018; Krause et al., 2019). *Enabling Self‐management and Coping with Arthritic Pain using Exercise*, *ESCAPE‐pain*, is a rehabilitation programme of education and exercise that reduces pain, improves mobility, physical and mental health and wellbeing (Hurley et al., 2010, 2012). Until recently *ESCAPE‐pain* was delivered by physiotherapists in hospital outpatient departments. Unfortunately, the financial, logistical and workforce constraints on health systems limits delivery of the programme. Delivering *ESCAPE‐pain* in community venues (leisure centres, community halls, etc) facilitated by exercise professionals, could increase access for many more people and reduce costs (Hurley & Carter, 2016).

As part of a Sport England initiative to increase physical activity in older people *ESCAPE‐pain* was delivered by community‐based organisations. We explored the experiences of the organisations and facilitators delivering the programme to understand what enables, impedes or prevents delivery, and what is required to sustain its delivery.

## 2 METHODS

*ESCAPE‐pain* is a rehabilitation programme for people with knee, hip and/or back pain, that integrates information, advice and support, with a progressive, challenging exercise regimen. It helps participants understand their problem, dispels erroneous health beliefs, advises them what (not) to do, enables them to experience the benefits of exercise and control of their symptoms. Detailed descriptions of the programme are available [(17) www.ESCAPE‐pain.org], but briefly *ESCAPE‐pain* is delivered to groups of 8–12 people, aged 45 years and older who attend 12 sessions (twice a week for 6 weeks) led by a trained facilitator. Each session comprises:

- a ∼25 min **education** component that takes the form of a themed discussion (covering causes of joint pain, prognosis, advice, and pain self‐management/coping strategies, such as heat/ice, rest‐activity cycling, relaxation) with behavioural change techniques (goal‐setting, action/coping planning, positive feedback, etc) threaded into the programme, and emphasises that exercise is a safe, effective way to reduce pain and increase function;
- a ∼40 min supervised **exercise** component where participants undertake a personalised, progressive exercise regimen to increase strength, endurance and function.

The blend of information, support, shared learning and experiential learning alters people's beliefs about joint pain, its impact and encourages adoption of healthier lifestyles (Hurley et al., 2010).

In 2017, Sport England's “*Active Ageing*” initiative called for programmes that could increase physical activity in older (defined by them as 55 years or over) inactive people (defined as people taking part in less than 30 min of physical activity per week). *ESCAPE‐pain* was accepted as a potential programme. Its entry criteria was adapted to align with the “Active Ageing” initiative age and inactivity criteria, but otherwise the format and content of the programme was unaltered from that described above. 17 leisure and community organisations collaborated with us to deliver 200 *ESCAPE‐pain* programmes across 75 sites. As an incentive the delivery organisation received a payment (£128) to cover the costs of delivering the programme for each person they recruited who was 55 years or older, “inactive” (doing less than 30 min physical activity per week) and returned outcome data (pain, function, quality of life, physical activity levels) when they started the programme, immediately after completing the programme, and 3‐, 6‐ and 12 months later. Facilitators were exercise professionals with Level 3 Exercise Referral qualifications, 150 h of experience and experienced in supervising people with health conditions (exercise on referral, cardiac or pulmonary rehabilitation programmes). All facilitators attended a 1 day training course that enabled them to deliver *ESCAPE‐pain*.

*Interviewees*. From the collaborating organisations 17 managers and 10 facilitators were contacted, all of whom agreed to be interviewed. The organisation managers comprised 10 organisations that serviced a specific urban town or city population, five organisations that served local rural populations across a geographic region or county and two national organisations that served several urban and rural populations across the England. Most of the organisations delivered *ESCAPE‐pain* in leisure centre gyms, but a few delivered the programme in local community halls.

*Data collection and analysis*. Semi‐structured telephone interviews (Appendix) were conducted, recorded, transcribed verbatim, coded using NVivo software, and thematic analysis identified emergent themes (Braun & Clarke, 2006). Managers were asked about their motivations for wanting to deliver the programme, practical issues they encountered, their opinions of the programme and plans for sustaining the programme. Facilitators were asked their experiences of training, programme deliver, feedback from participants and support they received from their organisations and external agencies. The interview schedules were adapted as necessary to ensure relevance to the interviewee. Comments have been anonymised to indicate the organisation or facilitators (Org1, Org2, Facil1, Facil2, etc.).

### 2.1 Findings

The findings relate to organisation's reasons for wanting to get involved with the programme, the practicalities and barriers people experienced implementing it, participant's feedback and plans for generating revenue to sustain delivery of the programme.

### 2.2 Setting up the programme

Leisure organisations were keen to be involved in Sport England initiatives and because *ESCAPE‐pain* was chosen to be included in their “Active Ageing” initiative the programme was seen as being endorsed by Sport England. Moreover, the programme aligned with many organisations plans to deliver more healthcare interventions. In addition, organisations appreciation of the importance of helping people with joint pain from their experiences of exercise referral schemes, collaborations with clinical departments and personal experiences. Programme facilitators were required to attend a one‐day training course, and although this incurred training, time, travel and sometimes accommodation costs, it was considered valuable staff development:

“…it's also been good for the development of the staff…there's been a lot of learning through as part of the co‐delivery, and shadowing, so I think that's helped them self‐develop...” (Org24)

Recruiting participants onto the programme was achieved through a variety of promotional activities they ran among their members, local press and social media. Recruitment was most successful where organisations formed collaborative partnerships with local GPs, physiotherapy departments and relevant clinical services, but these partnerships took time and effort to establish:

“…we're everywhere, we're all over the city, we're constantly at neighbourhood meetings, GP meetings, CCG meetings…it's taken us like what 18 months, to get to the point where we're at …” (Org7)

Leisure organisations delivering healthcare programmes were sometimes viewed with suspicion by local clinical services. They were perceived as lacking the necessary experience and expertise to treat people with “medical conditions”, and as potential competitors who could undermine local clinical services. Such fears dissipated when clinicians realised the exercise professionals were specially trained to deliver *ESCAPE‐pain*, and as trusting collaborative partnerships developed between clinical and leisure organisations:

“…we had a bit of backlash off physios, because they felt as though we were doing their job … Once we'd gone out, and we'd talk to them, and the CCGs had supported us on that, fine, absolutely fine...” (Org6)

Ensuring participants met the inclusion “clinical” criteria (knee or hip pain for more than 3 months, clinical diagnosis of OA, no unstable mental or physical health condition that prevented exercise) was straightforward. The additional criteria the “Active Ageing” initiative required (people doing less than 30 min of physical activity per week) was more challenging. In particular, one outcome (the Active Life Questionnaire) asked about breathlessness as an indication of physical inactivity, which confused people who usually attributed breathlessness to cardiovascular and respiratory co‐morbidities, rather than inactivity:

“…when you get to the breathlessness element of [Active Lives Questionnaire], they say ‘Well, every time I get out of the chair I'm breathless'...” (Org25)

The small financial incentive covered some, but not all, of their costs. Moreover, organisations did not receive this incentive if participants did not meet the inactive criterion, this created a dilemma for them of having to turn away people whose interest and hopes they had raised, so they often absorbed the programme costs of people who did not reach the entry criteria in the hope that it might ultimately benefit their organisation:

“…you don't want to be saying, 'No,' to people, and you don't, but all the time you're not saying, 'No,' … you're losing money…” (Org14)

### 2.3 Practicalities of delivering ESCAPE‐pain

Undertaking *ESCAPE‐pain* in a community setting was seen as being more convenient than attending an outpatient department and “de‐medicalised” joint pain:

“…[participants] don't want to be in a clinical setting…they see the hospital for the bad stuff, and this is more the fun stuff…” (Org1)

Others thought joint pain was a medical problem and should be treated in a medical setting:

“…when we held the courses at a medical facility…there just seemed to be a lot more positive response to that and I don't know if that's just because they trust you a bit more…” (Org4)

Exercise professionals were keen to highlight their expertise and their ability to support people with health conditions long term. The training programme improved their confidence in managing people with joint pain encouraging them to exercise, but they were not confident advising people about medication and referred questions about medications to a healthcare professional. In some instances a healthcare professional co‐delivered sessions that covered medication:

“…as a fitness instructor we're used to using that motivational interview, and we're used to talking to people as a group. We've got places for them to follow on afterwards … we need a little bit more support though, with that medication arm of it...” (Facil26)

Limited availability of exercise facilities (gyms, studios, etc) meant the programme was usually scheduled outside peak times, to avoid impinging on more lucrative programmes, and maximised the use of the venues and their resources:

“*… we just have to fit in with the leisure timetable…*” (Org24)

In fact older people often preferred using leisure facilities at quieter times when they felt less intimidated by younger gym users, and it was easier for them to use public transport. Adequate car parking availability was important in the recruitment of older people who were in pain and had limited mobility:

“…quite a few people dropped out… they haven't got a car park yet, so that was a bit of a problem because obviously people with osteoarthritis don't want a long walk…” (Org4)

For providers in rural areas, venue hire, travel and travel time were additional costs they needed to cover:

“…we've gone through more of the village hall, community settings … Our county's quite big … it can be an hour's drive each way, an hour of delivery, quarter of an hour, 20 minutes, either side of that sorting things out. That's a lot of man‐hours to deliver...” (Org25)

Collecting “clinical” outcomes at the five assessment timepoints (immediately before, after, 3, 6 and 12 months after the end of the programme) was onerous and increased the organisational workload:

“With our current workload and then doing this on top and also having to chase up the people on a regular basis to try and get the data in … It's quite a lot to do.” (Org11)

This was exacerbated by participants needing help to understand the questionnaires, and an explanation of the results to them:

“…you actually need to sit there and go through the forms individually with them, because they don't understand the language … The first session is practically a write‐off because it's just filling out forms...” (Org7)

“*…somebody needs to sit down and help people like us leisure trusts work out what those results mean…*” *(*Org6*)*

Despite these issues the organisations appreciated the need to demonstrate the programme's success and benefits to convince commissioners to fund the programme:

“…because of the evidence that it gathered we've been able to have really robust conversations with funders…” (Org1)

## 3 *ESCAPE‐PAIN*'s ETHOS AND STRUCTURE AND BENEFITS

The programme provided participants with information and advice they should have received, especially about the importance of physical activity in reducing joint pain (National Institute for Health & Clinical Excellence, 2014; Rausch Osthoff et al., 2018), to help people to gain confidence and start exercising:

“…we've been surprised how many people have been told not to do anything…we've then got to try and say, 'No, you need to keep moving, you need to keep doing these exercises...'” (Facil25)

Its informal format meant people with mixed abilities exercised together at their own pace, which engendered group cohesion and confidence to try things people were previously wary about:

“…for the people that wouldn't normally exercise, I think that's a brilliant introduction to it because it's not as frightening for them as say going in a gym … Even though it's a timed circuit, they can still go at their own pace. They do as much as they can in that time...” (Facil6)

Reduction in pain and improvement in physical function enabled people to reduce their reliance of analgesia and walking sticks and resume previous activities:

“…the majority of people have actually resumed things that they were doing before, like gardening, walking, outdoor stuff. Levels of activity of daily living have improved…” (Org9)

The social aspects and group interaction were a vital part of the programme's success as participants learnt to take control and self‐manage their problems.

“…the group atmosphere enables an expert to facilitate [participants] to elicit learning for themselves which becomes much more powerful when someone comes up with their own solutions as opposed to being told the solution…” (Org14)

Group delivery showed participants that they were not alone, encouraged them to share of ideas and experiences, which helped build bonds and friendships between the participants:

“…they find out [they are] not the only one who's got this level of frustration or this pain, or this feeling of being upset or feeling of lack of progress. Some feel isolation...” (Facil13)

Friendships developed between participants during a programme that often continued afterwards:

“…some of them want to continue as a group and that's because they've really enjoyed the group aspect and they've made some friends in the group….” (Org3)

Facilitators became fervent supporters of the programme because of the enthusiastic feedback from the participants:

“…I'm a really big advocate of [ESCAPE‐pian]. I see first‐hand that it literally does improve people's lives, improve their confidence, their mental well‐being…not only physically does it benefit them, it gives them almost a social aspect, all around mental health...” (Facil26)

To sustain the benefits of *ESCAPE‐pain* facilitators signposted participants to groups, classes and activities that might engage them and help them remain active. Facilitators took the opportunity to introduce participants the gym and equipment to reduce the fear, anxiety and intimidation older people often feel about leisure facilities:

“*…it makes sense obviously that we'*re *in there, we'*re *in a room down the corridor from the gym, let's go and have a peek in the gym, let's introduce you so it's not such a scary proposition…*” (Facil13)

Some venues developed activities and introduced programme participants to gym staff and facilities to support programme participants worried about attending a gym:

“…when we said come to the gym, 'Oh, I don't like going to the gym', so I do a class and now I've actually got people from my rehab coming into that one as well...” (Facil6)

Other participants were reluctant to attend a gym, and preferred supervision by a healthcare professional:

“...one of the participants said ‘I only want to come [to physiotherapy service] because we need to have the experts around’...” (Org5)

Alternative options were considered for people who did not want to use a gym.

“…it's just having a variety of options so they can continue to do…” (Org3)

Several organisations made brief videos of participants describing the benefits they had obtained from *ESCAPE‐pain*, which were used to raise awareness of local programmes and to encourage people to join one (https://ESCAPE‐pain.org/personal‐stories)

## 4 SUSTAINING THE PROGRAMME

The greatest barrier to sustaining delivery of *ESCAPE‐pain* was finance. As commercial organisations they had to cover administration, salaries, training, travel costs, venue, use of a room could be used for more profitable activities, etc.

“…it's perceived that these venues are free because they're in the leisure sector, but they're not because you could have a class being delivered in that room which is making money as opposed to this class which isn't…” (Org14)

Organisations did not profit from delivering *ESCAPE‐pain*, but saw its personal, professional, organisational and social value. All of them wanted to continue to deliver *ESCAPE‐pain* but to do this they needed to generate enough revenue to cover the delivery costs. To do this some organisations charged nominal and often subsidised membership fees or for participating on the programme, developed “post *ESCAPE‐pain*” classes to support people remain active and generated revenue from refreshments and merchandise:

“…that long term movement into other areas of the business, so whether that's joining one of our [branded] sessions or one of our health programmes and then becoming a member or as a pay as you go participant … how many people actually complete the programme and then go on to continue exercising with us would be a factor…” (Org13)

Forming clinical‐community partnerships were considered the best way to fund the programme long‐term. In a few places leisure organisations had been contracted by local clinical commissioners to deliver *ESCAPE‐pain* reducing costs and freeing NHS facilities. Data was essential in forming a convincing business case showing *ESCAPE‐pain* was needed, popular (had good uptake and retention), beneficial and reduced healthcare resources:

“…I had an annual report from ESCAPE‐pain just so the CCG could see it, how many people came to the doors, what the outcomes were … sometimes people just need in in black and white, they'll be governed by the money side of that…” (Org3)

## 5 DISCUSSION

This study showed that *ESCAPE‐pain* could be delivered in community venues by exercise professionals, and people reported very positive experiences. The main challenges to running the programme in the community was raising awareness that the programme was available locally, could be accessed by self‐referral, getting healthcare systems to support leisure organisations delivering a “healthcare intervention” and collecting outcomes. Many of these challenges could be overcome by forming partnerships with local healthcare commissioners and providers and deliver mutual benefits.

The Sport England *Active Ageing* initiative demanded recruiting people doing less than 30 min physical activity per week using a lengthy, complex outcome measure that people found difficult to understand and onerous to complete. It required people differentiate between breathlessness caused by performing physical activity and breathlessness caused by common comorbidities such as cardiorespiratory conditions, (I‐Min Lee et al., 2012; Sparling et al., 2015). People found this differentiation very difficult. Recruitment onto a “typical” *ESCAPE‐pain* programme is much easier as there is no (in)activity criteria, and only two short easy‐to‐complete outcomes are collected using an online system to minimise the burden. The data enables participants to gauge their progress, demonstrates to commissioners that after training exercise professionals can safely deliver high quality “healthcare interventions” and that the community‐based programme was as effective with outcomes comparable to those achieved in clinical settings (Hurley et al., 2018; Hurley, Walsh, Mitchell, Pimm, Patel, et al., 2007).

Once on the programme participants reported similar benefits to participants who attended *ESCAPE‐pain* programmes delivered by physiotherapists in hospital departments (Hurley et al., 2010). Community organisations and facilitators could see the benefits people were attaining from the programme and they wanted to continue to deliver *ESCAPE‐pain* after the “Active Ageing” initiative ended. To do so they needed to generated revenue to recover their delivery costs. This was achieved by some organisations charging the full cost of the 12 session programme (between £24‐£60), usually based on charges for similar rehabilitation or exercise‐on‐referral programmes, and often included use of the centre's other leisure and social facilities and activities (swimming, yoga, exercise classes, etc). Some used the programme to attract new members, sometimes at reduced‐rates. The sale of refreshments, food, merchandise, etc, was another source of new income. Others developed “post‐programme” activities to support participants remain active retaining the benefits that clinical departments cannot offer. Although these are additional out‐of‐pocket expenses that people have to pay for, people are willing to pay for effective interventions that reduce pain and its impact (Hurley, Walsh, Mitchell, Pimm, Williamson, et al., 2007; Kotlarz et al., 2009; Puig‐Junoy & Ruiz Zamora, 2015).

An alternative way to sustain delivery of the programme is for health systems to contract community providers to deliver the programme. Managing the millions of people suffering knee and hip OA is one of the largest areas of healthcare utilisation and expenditure. Healthcare providers struggle to meet this demand due to financial, logistic and workforce limitations, which have been severely exacerbated by the COVID19 pandemic. Community providers have greater capacity to meet this demand and relieve the burden on health systems. They can also provide many opportunities for people to habitualise regular physical activity after completing *ESCAPE‐pain*, thereby retaining the benefits attained. The NHS “Long Term Plan” aims to establish “Integrated Care Systems” to deliver safe, effective healthcare outside hospitals in people's local community where it is easier to access (NHSE, 2019). Two of our community organisations have formed partnerships with local healthcare commissioners to deliver *ESCAPE‐pain* to help them address the massive unmet demand, which generates opportunities for the community providers to expand their involvement in healthcare. This makes financial sense as the cost of running the programme in NHS outpatient departments is estimated to be about £400 per person, much higher than community providers due to higher estate costs and salaries (Curtis, 2019). Public Health England estimated delivering *ESCAPE‐pain* in hospital outpatient departments yields a return of £5.20 for each £1 invested (Public Health England, 2017), while a report from the York Economic Health Consortium commissioned by the NHS Innovation Accelerator estimated community‐based *ESCAPE‐pain* has a return on investment of £8.80 for every £1 invested (York Health Economics Consortium, 2019).

*Strengths and limitations*. The strengths of the study are its size, representativeness and generalisability. It was a sizable qualitative study that gathered the opinions of a relatively large number of different types of community organisations, such as large national leisure organisations, local authority run enterprises and smaller local charities. This provides an evidence base and case studies representative of the types of community organisations who might want to replicate the programme and apply it to their specific context and might be transferable to similar health programmes other than *ESCAPE‐pain*.

However, we don't have information from organisations who discontinued the programme, who's experiences are likely to be less positive. A facilitator from an organisation who discontinued the programme was interviewed, and the organisations who had implemented the programme were honest in describing the challenges they encountered. Most of the organisations were urban‐based, which will skew the challenges and solutions experienced in urban settings. We did capture specific issues faced by a few rural‐based organisations, but more data would have been useful.

In summary, *ESCAPE‐pain* can be delivered by exercise professionals in community settings safely, effectively, and efficiently. This benefits people suffering joint pain who get faster, easier access to better care, with greater opportunities for on‐going support. It enables health commissioners to manage the huge and increasing demand more efficiently, savings resources. In addition, fostering partnerships between local health stakeholders and community providers fulfils national policy of encouraging self‐management of long‐term condition in the community, provides community providers with new business opportunities and enables them to contribute to improved health, wellbeing of their local population.

The COVID19 pandemic has made improving access to effective healthcare vital. Establishing *ESCAPE‐pain* as a community‐based programme makes it more accessible to people who need it, when they need it, reducing the logistic and financial burden on healthcare systems, and helping people to live better and do more. This would be welcomed by millions of people living with joint pain.

## CONFLICT OF INTEREST

The authors report no conflicts of interest.

## AUTHOR CONTRIBUTIONS

Michael Hurley: Conceived and obtained for the study; supervised running and conduct of the study; supervised analysis; led preparation of all drafts and final manuscript; custodian of the data; contact author. Helen Sheldon: Oversaw running of the study; contributed to analysis; involved in preparation of drafts and final manuscript. Margaret Connolly: Oversaw running of the study; contributed to analysis; involved in preparation of drafts and final manuscript. Andrea Carter: Conceived and obtained for the study; supervised running of the study; contributed to analysis; involved in preparation of drafts and final manuscript. Rachel Hallett: Conducted interviews and collected data; led analysis; involved in preparation of drafts and final manuscripts.

## ETHICS STATEMENT

As this was an evaluation of an existing programme ethical approval was deemed not to be required. The reason we were conducting the study was explained to all interviewees, and it was emphasised they had the right to refuse to be interviewed, withdraw from an interview and/or ask for their interview to be deleted at any time.

## Data Availability

Data sharing not applicable to this article as no datasets were generated or analysed during the current study.

## Appendix

**Community provider organisations**

Need for the programme

- Why did you decide to opt for and roll out *ESCAPE‐pain*?
- What information and evidence was required to initiate the process?
- Who were the key people involved in the process?

Operationalising the programme

- Describe how *ESCAPE‐pain* was put into practice by your organisation
  → How it was staffed?
  → Where it was held?
  → What systems had to be prepared/adapted/introduced?
  → Was the programme adapted? Why? How?
  → What helped/hindered implementation? Why? How overcome?
- What were the main problems you encountered during implementing the programme?
- How were these overcome?
- How was *ESCAPE‐pain* evaluated?
- Are the commissioners happy with the programme?
- What are their criteria for success?

Sustaining and spreading ESCAPE‐pain

- What are your organisations future plans for *ESCAPE‐pain*?
- What will be the main barriers to sustaining the programme?
- Can these be overcome? How?

### Programme facilitators–exercise professionals

Training course

- How did you find the training?
  → Did it enable you to deliver the programme?
  → What's good and bad about the training?
- How should the training be changed to improve your ability to deliver the programme?

The ESCAPE‐pain programme.

What's good and bad about the *ESCAPE‐pain* programme?

## References

[msc1584-bib-0001] Braun, V. , & Clarke, V. (2006). Using thematic analysis in psychology. Qualitative Research in Psychology, 3, 77–101.

[msc1584-bib-0002] Cross, M. , Smith, E. , Hoy, D. , Nolte, S. , Ackerman, I. , Fransen, M. , Bridgett, L. , Williams, S. , Guillemin, F. , Hill, C. L. , Laslett, L. L. , Jones, G. , Cicuttini, F. , Osborne, R. , Vos, T. , Buchbinder, R. , Woolf, A. , & March, L. (2014). The global burden of hip and knee osteoarthritis: Estimates from the global burden of disease 2010 study. Annals of the Rheumatic Diseases, 73, 1323–1330. 10.1136/annrheumdis-2013-204763 24553908

[msc1584-bib-0003] Curtis, L. (2019). Unit costs of health and social care. Www.Pssru.Ac. UK. https://www.pssru.ac.uk/project‐pages/unit‐costs/unit‐costs‐2019/

[msc1584-bib-0004] Hunter, D. J. , & Bierma‐Zeinstra, S. (2019). Osteoarthritis. The Lancet. 393(10182), pp. 1745–1759. 10.1016/S0140-6736(19)30417-9 31034380

[msc1584-bib-0005] Hunter, D. J. , Schofield, D. , & Callander, E. (2014). The individual and socioeconomic impact of osteoarthritis. Nature Reviews Rheumatolology, 10(7), 437–441. 10.1038/nrrheum.2014.44 24662640

[msc1584-bib-0006] Hurley, M. , & Carter, A. (2016). ESCAPE‐into the community ‐ a community‐based rehabilitation programme for elderly people with chronic joint pain. Perspectives in Public Health, 136(2). 10.1177/1757913915626351 26933072

[msc1584-bib-0007] Hurley, M. , Dickson, K. , Hallett, R. , Grant, R. , Hauari, H. , Walsh, N. , Stansfield, C. , & Oliver, S. (2018). Exercise interventions and patient beliefs for people with hip, knee or hip and knee osteoarthritis: A mixed methods review. Cochrane Database of Systematic Reviews, Issue 4. Art. No.: CD010842. 10.1002/14651858.CD010842.pub2. Accessed 06 August 2021. 29664187 PMC6494515

[msc1584-bib-0008] Hurley, M. , Walsh, N. , Bhavnani, V. , Britten, N. , & Stevenson, F. (2010). Health beliefs before and after participation on an exercised‐based rehabilitation programme for chronic knee pain: Doing is believing. BMC Musculoskeletal Disorders, 11(1), 31. http://www.biomedcentral.com/1471‐2474/11/31 20149236 10.1186/1471-2474-11-31 PMC2828988

[msc1584-bib-0009] Hurley, M. , Walsh, N. E. , Mitchell, H. , Nicholas, J. , & Patel, A. (2012). Long‐term outcomes and costs of an integrated rehabilitation program for chronic knee pain: A pragmatic, cluster randomized, controlled trial. Arthritis Care & Research, 64(2). 10.1002/acr.20642 21954131

[msc1584-bib-0010] Hurley, M. , Walsh, N. E. , Mitchell, H. L. , Pimm, T. H. , Williamson, E. , Jones, R. H. , Reeves, B. C. , Dieppe, P. A. , & Patel, A. (2007a). Economic evaluation of a rehabilitation program integrating exercise, self‐management, and active coping strategies for chronic knee pain. Arthritis & Rheumatism, 57(7), 1220–1229. 10.1002/art.23011 17907207 PMC2675012

[msc1584-bib-0011] Hurley, M. , Walsh, N. E. , Mitchell, H. L. , Pimm, T. J. , Patel, A. , Williamson, E. , Jones, R. H. , Dieppe, P. A. , & Reeves, B. C. (2007b). Clinical effectiveness of a rehabilitation program integrating exercise, self‐management, and active coping strategies for chronic knee pain: A cluster randomized trial. Arthritis & Rheumatism, 57(7), 1211–1219. 10.1002/art.22995 17907147 PMC2673355

[msc1584-bib-0012] Kotlarz, H. , Gunnarsson, C. L. , Fang, H. , & Rizzo, J. A. (2009). Insurer and out‐of‐pocket costs of osteoarthritis in the US: Evidence from national survey data. Arthritis & Rheumatism, 60(12), 3546–3553. 10.1002/art.24984 19950287

[msc1584-bib-0013] Krause, V. B. , Sprowl, K. , Powell, K. E. , Buchner, D. , Bloodgood, B. , Piercy, K. , George, S. M. , & Kraus, W. E. (2019). Effects of physical activity in knee and hip osteoarthritis: A systematic umbrella review. Medicine & Science in Sports & Exercise, 51(6), 1324–1339. 10.1249/MSS.0000000000001944.Effects 31095089 PMC6527143

[msc1584-bib-0014] Lee, I.‐M. , Shiroma, E. J. , Lobelo, F. , Puska, P. , Blair, S. N. , & Katzmarzyk, P. T. (2012). Effect of physical inactivity on major non‐communicable diseases worldwide: An analysis of burden of disease and life expectancy. The Lancet, 380(9838), 219–229. 10.1016/s0140-6736(12)61031-9 PMC3645500 22818936

[msc1584-bib-0015] National Institute for Health and Clinical Excellence . (2014). Osteoarthritis: The care and management of osteoarthritis in adults . CG177. http://www.nice.org.uk/guidance/cg177 25340227

[msc1584-bib-0016] NHS England (2015). CCG programme Budgeting Benchmarking tool 2013/14). Office for National Statistics (ONS). Sickness Absence Report 2017). https://www.england.nhs.uk/prog‐budgeting/

[msc1584-bib-0017] NHSE . (2019). NHS long term plan. https://www.longtermplan.nhs.uk/

[msc1584-bib-0018] Public Health England . (2017). Return on investment of interventions for the prevention and treatment of musculoskeletal conditionsitle.

[msc1584-bib-0019] Public Health England . (2018). MSK now a priority. https://www.arthritisresearchuk.org/health‐professionals‐and‐students/network‐news/may‐18/msk‐now‐a‐phe‐priority.aspx

[msc1584-bib-0020] Puig‐Junoy, J. , & Ruiz Zamora, A. (2015). Socio‐economic costs of osteoarthritis: A systematic review of cost‐of‐illness studies. Seminars in Arthritis and Rheumatism, 44(5), 531–541. 10.1016/j.semarthrit.2014.10.012 25511476

[msc1584-bib-0021] Rausch Osthoff, A. K. , Niedermann, K. , Braun, J. , Adams, J. , Brodin, N. , Dagfinrud, H. , Duruoz, T. , Esbensen, B. A. , Günther, K. P. , Hurkmans, E. , Juhl, C. B. , Kennedy, N. , Kiltz, U. , Knittle, K. , Nurmohamed, M. , Pais, S. , Severijns, G. , Swinnen, T. W. , Pitsillidou, I. A. , & Vliet Vlieland, T. P. M. (2018). 2018 EULAR recommendations for physical activity in people with inflammatory arthritis and osteoarthritis. Annals of the Rheumatic Diseases, 77(9), 1251–1260. 10.1136/annrheumdis-2018-213585 29997112

[msc1584-bib-0022] Sparling, P. B. , Howard, B. J. , Dunstan, D. W. , & Owen, N. (2015). Recommendations for physical activity in older adults. BMJ, 350, h100. 10.1136/bmj.h100 25608694

[msc1584-bib-0023] Versus Arthritis (2014). Musculoskeletal health – a public health approach. https://www.versusarthritis.org/policy/policy‐reports/musculoskeletal‐health/

[msc1584-bib-0024] Versus Arthritis (2019). The State of Musculoskeletal Health ‐ Arthritis and other musculoskeletal conditions in numbers.

[msc1584-bib-0025] Vos, T. , Lim, S. S. , Abbafati, C. , Abbas, K. M. , Abbasi, M. , Abbasifard, M. , Abbasi‐Kangevari, M. , Abbastabar, H. , Abd‐Allah, F. , Abdelalim, A. , Abdollahi, M. , Abdollahpour, I. , Abolhassani, H. , Aboyans, V. , Abrams, E. M. , Abreu, L. G. , Abrigo, M. R. M. , Abu‐Raddad, L. J. , Abushouk, A. I. , & Murray, C. J. L. (2020). Global burden of 369 diseases and injuries in 204 countries and territories, 1990‐2019: A systematic analysis for the global burden of disease study 2019. The Lancet, 396(10258), 1204–1222. 10.1016/S0140-6736(20)30925-9 PMC7567026 33069326

[msc1584-bib-0026] York Health Economics Consortium . (2019). Economic evaluation case study: ESCAPE‐pain. https://nhsaccelerator.com/wp‐content/uploads/2020/12/NIA‐Case‐Study‐ESCAPE‐pain‐FINAL‐22.08.19.pdf

